# ESWT has stood the test of time

**DOI:** 10.1097/JS9.0000000000003823

**Published:** 2025-10-29

**Authors:** Kandiah Raveendran

**Affiliations:** Department Orthopedic Surgery, Fatimah Hospital, Ipoh, Malaysia

In 2015, the *International Journal of Surgery* published a Special Issue on Shockwave treatment^[1]^ with the assistance of the International Society for Medical Shockwave Treatment (ISMST).

I was the editor for that Special issue, and I wrote the editorial titled “ESWT is a force to be reckoned with”^[2]^. This issue had 20 papers, mostly review papers, and all the articles were invited from experts in the field of shockwave medicine. This special issue was well reviewed and cited.

In 2024, I mooted the idea of a second Special issue, but this time we opened it to all members of the ISMST, as I wanted original articles. ESWT is now a worldwide “phenomenon” with shockwave societies in many countries. The research in ESWT has multiplied over the last 10 years, including basic science and clinical research with many high-quality studies. The clinical indications have also widened with ESWT being used for cardiac and neurological diseases.

The *International Journal of Surgery* has also evolved over the last 10 years into an extremely reputable journal with a high impact factor ranking second among all surgical journals. All submissions were subjected to a rigorous editorial process, and only 13 articles were accepted. There are five basic science articles and eight clinical studies.

The working mechanism of shockwave has been studied extensively. Othmar Josef Wess^[3]^ developed a model on momentum transfer at the different layers of biological tissue. He concluded that this mechanism is the basis for mechano-transduction and mechano-sensory-transduction.

Fan Hu *et al*^[4]^ used nano-motor composite microneedles as a transdermal delivery system for zoledronic acid. The shockwaves were used to deliver zoledronic acid and calcium to low bone density areas to improve the bone density. This study was done in ovariectomized mice. This novel transdermal system could reduce the systemic side effects of intravenous zoledronic acid as well as to target low BMD areas if clinically applied in the future.

This third article by Zong-Sheng Wu *et al*^[5]^ investigated the mechanism by which low-energy shockwaves can improve cystitis in a rat model. miRNAs are involved in bladder inflammation, and low-energy shockwaves can mitigate their effects. ESWT has been clinically used for interstitial cystitis/bladder pain syndrome in patients. The findings in a rat model also showed that shockwaves can improve intravesical drug retention.

Jiunn-Jye Sheu *et al*^[6]^ showed that pretreatment of mesenchymal stem cells with shockwaves could improve left ventricular ejection and inhibit left ventricular remodeling in mini-pigs with old myocardial infarction. Elaborate in vivo and in vitro studies showed angiogenesis in shockwave-treated animals with improved cardiac function as compared to the control group of mini pigs.

Spinal cord injury is a debilitating condition without any effective treatment. Jai Hong Cheng *et al*^[7]^ have used EWST in the rat model showing significant improvements in motor recovery, tissue regeneration, anti-inflammatory effects and mitochondrial protection. A multi-center prospective randomized trial is still ongoing and results are awaited.^[8]^

Shu-Jui Ko *et al*^[9]^ studied the combination of subacromial hyaluronic acid (HA) injection with ESWT in treating rotator cuff lesions without complete tears. Patients were divided into three groups, and an MRI was done before the study and 12 months later. ESWT provides additional benefits when combined with HA injections for patients with partial rotator cuff lesions.

A prospective study on the use of ESWT for calcific tendinitis of the shoulder improved symptoms, reduced calcification, enhanced tissue perfusion and promoted angiogenesis and BMP7 activity. Jai Hong Cheng *et al*^[10]^ looked at both radiolucent and radio-dense calcifications and reinforced ESWT as an effective treatment for calcific tendinitis of the shoulder.

Jakub Katolicky *et al*^[11]^ in a prospective study studied 21 athletes with chronic patella tendinopathy. ESWT was applied over four weekly sessions. Ultrasound evaluation showed improved results in clinical symptoms and tendon structure.

A prospective randomized study by Tomas Nedelka *et al*^[12]^ was conducted in 128 patients with chronic lumbar facet syndrome. Patients were randomized to receive ESWT or sham therapy. They were reviewed at 6 and 12 months, with a significant reduction in VAS scores. MRI done showed a reduction of bone marrow edema in the treated group as opposed to the sham group. There were no adverse effects reported.

Yoon Soo Cho *et al*^[13]^ investigated the effect of ESWT on the skin microbiome of burn patients. This retrospective study on 19 patients with burn scars who were treated with ESWT weekly for 3 months showed that ESWT enhances microbial diversity and modifies the microbial community structure in burn scars. This contributes to improved skin health and recovery aiding scar remodeling.

The use of ESWT in spastic cerebral palsy has been well documented. This meta-analysis by Thijs Wim Janssen *et al*^[14]^ looked at 12 randomized controlled trials. The combined study population included 421 children. ESWT showed positive effects across multiple parameters in children with spastic cerebral palsy.

Wen-Yi Chou *et al*^[15]^ studied the use of ESWT in femur and tibia nonunion in 91 patients. This study continues to confirm the role of ESWT in the treatment of nonunion. Bony union was achieved in 62.6% of patients with just one session of ESWT. I also confirmed, as in previous studies, that atrophic nonunion, larger fracture gaps and multiple surgeries before ESWT are associated with poorer outcomes.

Soo Young Joo *et al*^[16]^ did a double blind randomized controlled study on 120 patients with post-burn nerve injury and hypertrophic scars. The study showed more significant improvements in the ESWT group than in the sham group as regards VAS score, extension ROMs of hand joints and skin characteristics. This improved hand function, improved scarring, and alleviated pain.

## Conflicts of interest disclosure

There are no conflicts of interest.
